# Characterization of the Molecular Alterations Induced by the Prolonged Exposure of Normal Colon Mucosa and Colon Cancer Cells to Low-Dose Bisphenol A

**DOI:** 10.3390/ijms231911620

**Published:** 2022-10-01

**Authors:** Vidhya A Nair, Lara J Bou Malhab, Wael M. Abdel-Rahman

**Affiliations:** 1 Sharjah Institute for Medical Research, University of Sharjah, Sharjah 27272, United Arab Emirates; 2 Department of Medical Laboratory Sciences, College of Health Sciences, University of Sharjah, Sharjah 27272, United Arab Emirates

**Keywords:** bisphenol A, colon cancer, colon epithelial cells, DNA damage, invasion, next-generation sequencing, protein kinases

## Abstract

Colorectal cancer is a common cancer with a poor prognosis in both males and females. The influence of bisphenol A (BPA), a widely used environmental contaminant, in colon cancer development and progression is not well identified, in spite of the fact that the most common mode of exposure to BPA is ingestion. The aim of this work is to elucidate the carcinogenic effects of BPA in the colon in vitro. We analyzed BPA’s effects on human colon epithelial (HCoEpiC) and colon cancer (HCT116) cells. BPA exerted cytotoxic effects and augmented the 5FU cytotoxicity on both cell lines at high doses, while it did not show this effect at low doses. Therefore, we focused on studying the effects of low-dose (0.0043 nM) exposure on normal colonic epithelial cells for a long period of time (two months), which is more consistent with environmental exposure levels and patterns. BPA increased cellular invasiveness through collagen and the ability to anchorage-independent cell growth, as measured by colony formation in soft agar, which could support oncogenicity. To gain insights into the mechanism of these actions, we performed transcriptomic analysis using next-generation sequencing, which revealed 340 differentially expressed transcripts by BPA in HCT116 and 75 in HCoEpiC. These transcripts belong in many cancer-related pathways such as apoptosis, cell proliferation, signal transduction, and angiogenesis. Some of the significant genes (*FAM83H*, *CXCL12*, *PITPNA*, *HMOX1*, *DGKZ*, *NR5A2*, *VMP1*, and *ID1*) were confirmed by quantitative RT-PCR. Furthermore, BPA induced the phosphorylation of protein kinases such as JNK1/2/3, GSK-3α/β, AMPKα1, AKT1/2/3, AMPKα2, HSP27, β-catenin, STAT2, Hck, Chk2, FAK, and PRAS40 in HCoEpiC, as well as GSK-3α/β, p53, AKT1/2/3, p70 S6 kinase, and WNK1 in HCT116. The majority of these proteins are involved in potential carcinogenic pathways. Taken together, these data suggest that BPA plays a role in colon carcinogenesis, and they provide insights into the molecular mechanisms of colon epithelial cell transformation by BPA. Increasing exposure to environmental toxins such as BPA can explain the increasing incidence of colorectal cancer.

## 1. Introduction

Cancer is a notorious disease that features on the World Health Organization’s lists of the “top ten leading causes of death”, particularly in the upper-middle and high-income economy groups. This has been hypothetically explained by the increased longevity of these groups since cancer is considered to be a disease of the elderly. Aging offers the chance to accumulate the DNA damage and oncogenic mutations that are required for cancer development. An emerging challenge which hinders the application of universal management protocol in many cancer types is the heterogeneity of the disease presentation and outcome that is observed worldwide in different locations and ethnicities [1,2,3]. Moreover, in spite of the major progress that has been achieved in our knowledge about cancer, almost half of the patients with, for example, colon cancer are still destined to die from their disease [4]. Cancer mortality is mainly due to metastasis, which might be present at the time of diagnosis. These issues highlight the need for more effective, evidence-based measures for cancer prevention. The epidemiological features, clinical presentation, molecular genetics, and epigenetic profiles, as well as the disease outcomes of many cancers, seem to be linked to environmental factors [1,2,5,6]. A good example of this is the increasing environmental exposure to bisphenol A (BPA), which can bind estrogen receptors (or mimic their actions) and, hence, can act as an endocrine disruptor chemical (EDC). BPA is a widely spread environmental contaminant that has been associated with breast and other endocrine-related cancers such as those of the prostate and ovaries [5,7]. BPA predisposes to these tumors because it can act as a xenoestrogen, and hence, it is a classical EDC. Many EDCs accumulate in various compartments of the environment including the air, water, and soil and may be present in foods of plant and animal origin. The most common source of exposure to BPA is the migration of this plasticizer from food and drink containers into the contents when heated or even under normal conditions of use [6]. BPA can exert stimulatory action in cancer cells via the G-protein-coupled estrogen receptor 1 (GPER1, formerly known as GPR30, or G-protein coupled receptor 30) to activate the epidermal growth factor receptor (EGFR)/extracellular signal-regulated kinase (ERK)1/2 signaling pathway [8]. BPA can damage DNA and interfere with many cancer-related signaling pathways [9,10]. Low doses of BPA can also induce resistance in many types of cancer cells to classical chemotherapeutics such as doxorubicin, cisplatin, carboplatin, tamoxifen (TAM), bevacizumab, PARP inhibitors, vinblastine, and other drugs both in vitro and in vivo [5].

The carcinogenic effects of BPA have been demonstrated in the context of hormone-related cancers such as those of the breast [7], ovaries [11,12], and prostate [13]. The orally ingested BPA comes in direct contact with gastrointestinal mucosal cells; therefore, it is likely to exert its toxic effects on the gut and enhance the transformation of the gastrointestinal cells. Indeed, BPA treatment has induced intestinal toxicity and disrupted the barrier function in mice [14]. BPA predisposes intestinal epithelial cells to oxidative stress by increasing reactive oxygen species (ROS) and reactive nitrogen species (RNS), as well as by decreasing the activity of antioxidant enzymes (SOD, GPx, CAT, and T-AOC). Moreover, BPA induces inflammatory responses via multiple mechanisms including upregulating the key factors of the innate immune system (TLR2, TLR4, MyD88, and NF-κB) and secreting pro-inflammatory cytokines (IL-1β, IL-6, IL-8, and TNF-α) [15]. BPA and its substitute, fluorene-9-bisphenol (BHPF), induced significant alterations in gut metabolites associated with the inflammatory response in a dextran sulfate sodium-induced colitis mouse model [16]. It is well-established that persistent chronic inflammation can result in cancer development, particularly in the colon [17,18]. Recent studies have shown that the urinary BPA levels of colorectal cancer patients were significantly higher than those of controls [19,20], and that the positive association between BPA and colorectal cancer might be partly mediated by the oxidative stress marker 4-hydroxy-2-nonenalmercapturic acid (HNE-MA) [21]. However, there are fewer reports which have analyzed the carcinogenic effect of low-dose BPA on colon mucosa as compared to those that have addressed these effects on the breast and other endocrine-responsive organs.

The current work aims to clarify the effects of low-dose BPA exposure on colon mucosa and colorectal cancer cells and whether or not it can transform colon epithelial cells. The data presented here clarify many of the molecular changes and signaling pathways altered by BPA in the colon that can lead to cellular transformation.

## 2. Results

### 2.1. BPA and Cell Viability

**Figure 1:**
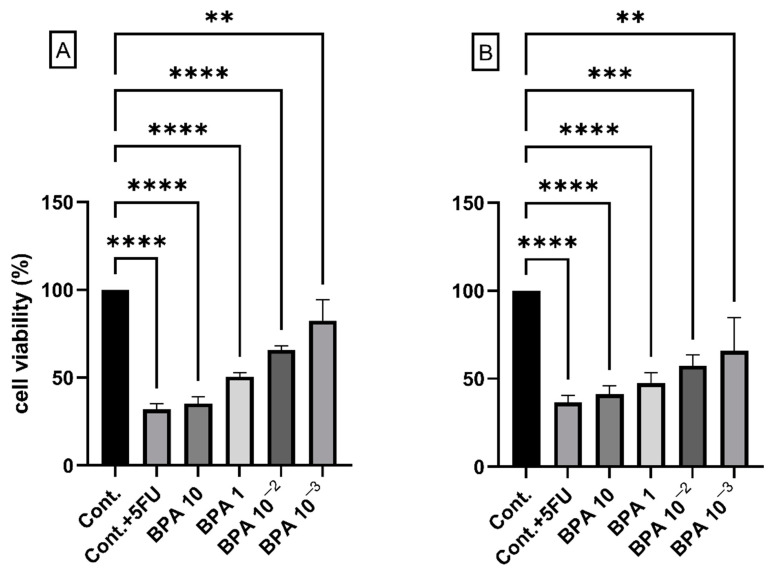
Representative graph showing the altered viability of (**A**) HCT116 and (**B**) HCoEpiC cells treated with decreasing concentrations of BPA (10, 1, 1.01, and 0.001 ug/mL) for 48 h. The control (cont.) was treated with the vehicle (DMSO) alone. The effect of overnight exposure to a therapeutic concentration of 5FU alone was used (second lane) as a reference. Data are shown as means ± SEMs, and ** *p* < 0.01, *** *p* < 0.001, and **** *p* < 0.0001. One-way ANOVA was followed by the Dunnett post-hoc test, with *n* = 3 (3 biological × 3 technical).

We first tested the effects of exposure to different concentrations of BPA on both the HCT116 colon cancer cell line and the normal colon epithelial cell line HCoEpiC. High concentrations of BPA (1 and 10 ug/mL) reduced viability, while exposure to a low concentration of 0.001 ug/mL (equivalent to 0.0043 nM) was the least effective at reducing cell viability (Figure 1). We focused on the low concentrations (0.0043 nM) to study the carcinogenic effects of BPA, which is more consistent with environmental human exposure values. The main focus of this work was to analyze the transformation potential of BPA on normal epithelial cells, which is a long process that takes a long time in vivo, therfore we opted for a prolonged exposure (2 months), after which we performed further functional analyses to dissect the potential long-term effects of BPA on normal colon mucosa cells (HCoEpiC). We used the same strategy to analyze the consequences of the continuous exposure of cancer cells (HCT116) to BPA to evaluate the effects of BPA on cancer progression.

### 2.2. BPA and Collagen Invasion

**Figure 2:**
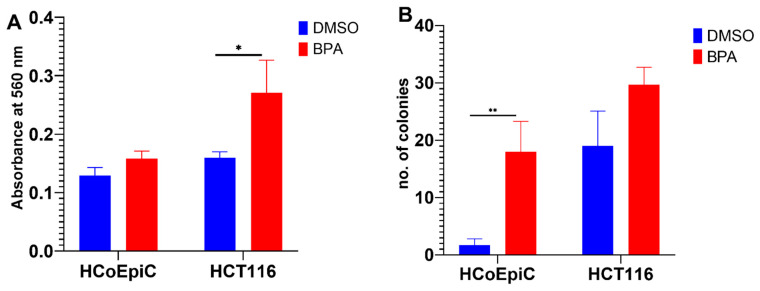
Cellular transformation after prolonged low-dose exposure to BPA. (**A**) Collagen invasion assay of HCoEpiC and HCT116 cell lines exposed to BPA for two months as compared to the base line invasion of the control (vehicle-exposed) cells. The bars represent the quantitative evaluation of cell invasion as measured by dye elution and spectrophotometric reading at 560 nm. (**B**) Colony formation in soft agar. HCoEpiC and HCT116 cell lines, exposed to BPA or the vehicle (DMSO) for two months, were seeded on soft agar in 6-well plates for 3–4 weeks. The cells were fixed and stained, and the number of colonies was counted in 5 × 100 power fields. The data shown are means ± SEMs, and * *p* < 0.05 and ** *p* < 0.01, with *n* = three biological replicates.

BPA increased cellular invasiveness to significant levels above the control in HCT116, while the increase in HCoEpiC did not reach statistical significance. This experiment was performed on the 2-months-exposed cells (Figure 2A).

### 2.3. Colony Formation in Soft Agar

The cell transformation detection assay is an anchorage-independent growth assay in soft agar and is considered to be a stringent assay for detecting the malignant transformation of cells in vitro. This experiment was performed on the 2-months-exposed cells. BPA increased the colony formation above the control level in both the HCT116 and HCoEpiC cell lines; the increase in HCoEpiC was statistically significant (Figure 2B).

### 2.4. Proteomic Analysis (Human Phospho Kinase Array)

**Figure 3:**
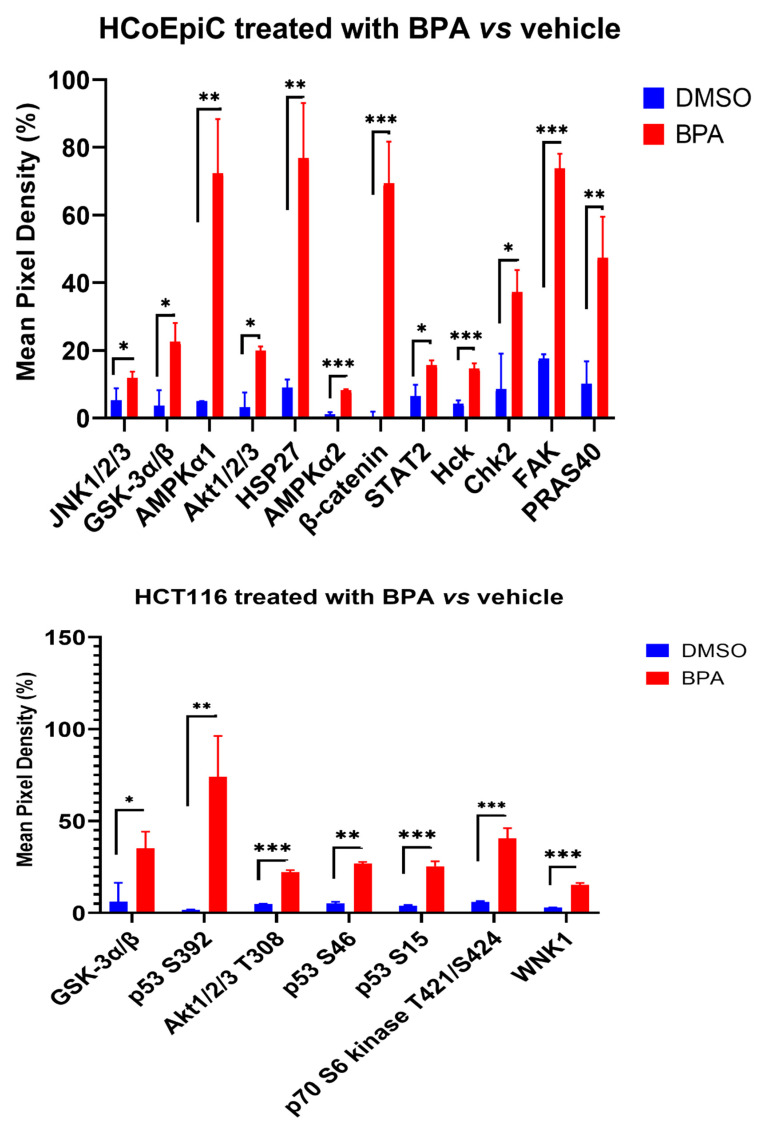
Phospho-kinase array analysis after exposure of the HCoEpiC and HCT116 cell lines to BPA for two months as compared to the vehicle (DMSO). Whole cell extracts were processed with the “human phospho-kinase array” ready-made kit. Data were analyzed using the “Image J” program and the means +/− the SEMs of the pixel density were blotted (in relation to the positive control spot). Out of the 43 tested proteins, only the proteins which showed a significant increase in the test compared to the control (DMSO) are shown. The data shown are means ± SEMs, and * *p* < 0.05, ** *p* < 0.01, and *** *p* < 0.001, with *n* = 3.

In the HCoEpiC cell line, there was a significant increase in 12 phosphoproteins: c-Jun N-terminal protein kinase (JNK1/2/3), glycogen synthase kinase 3 alpha/beta (GSK-3α/β), 5′-AMP-activated protein kinase catalytic subunit alpha-1 (AMPKα1), protein kinase B (PKB, AKT1/2/3), AMPKα2, heat shock protein 27 (HSP27), β-catenin, the signal transducer and activator of transcription 2 (STAT2), tyrosine-protein kinase HCK (Hck), checkpoint kinase 2 (chk2), focal adhesion kinase (FAK), and AKT1 substrate 1 (PRAS40), while in the HCT116 cell line, there was a significant increase in GSK-3α/β, tumor protein p53 (p53), AKT1/2/3, ribosomal protein S6 kinase beta-1 (S6K1) (also known as p70 S6 kinase), and lysine-deficient protein kinase 1/with-no-lysine kinase 1 (WNK1) (Figure 3). The majority of these proteins are involved in potential carcinogenic pathways in addition to their role in the response to toxic agents.

### 2.5. Next-Generation Sequencing

**Figure 4:**
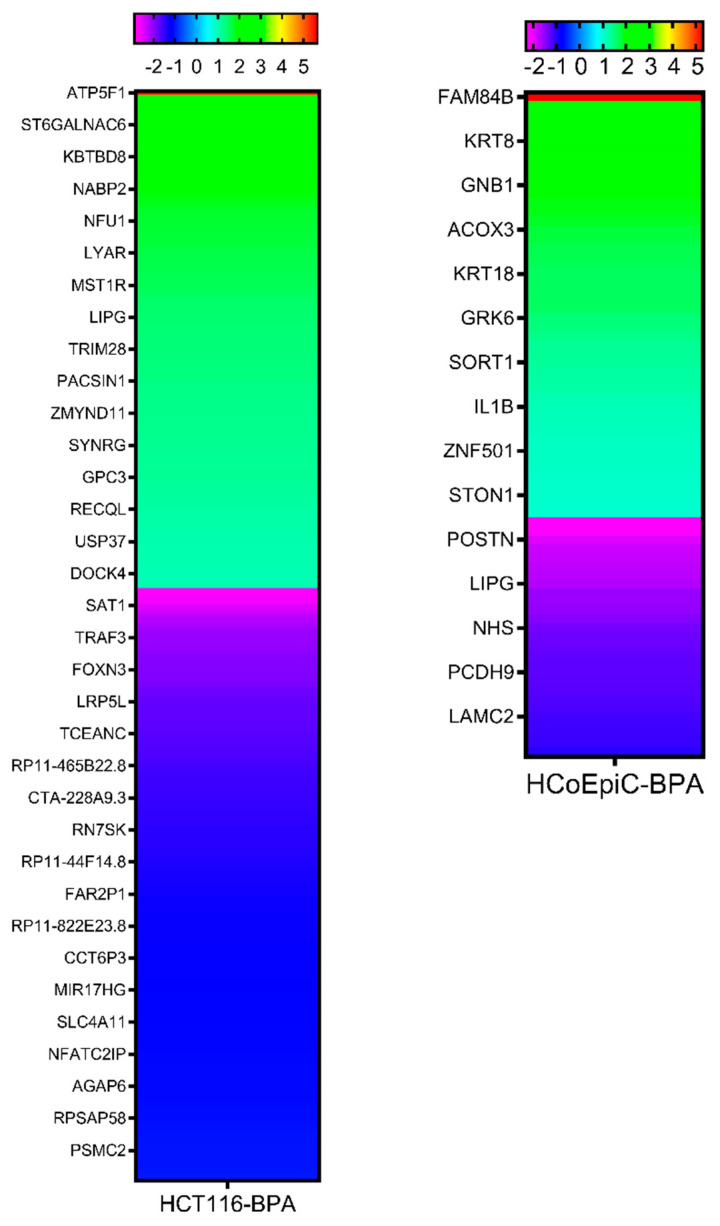
Heat map of the miRNA expression data obtained with NGS from the HCT116 and HCoEpiC cell lines treated with BPA for two months compared to vehicle-treated control. The relative miRNA expression is depicted according to the color scale shown above.

The next-generation sequencing (NGS) revealed 340 (155 upregulated genes and 185 downregulated genes) differentially expressed transcripts by BPA in the HCT116 and 75 (48 upregulated genes and 27 downregulated genes) in the HCoEpiC cell lines (Figure 4). The full lists can be found in Supplementary Tables S1 and S2. The data have been deposited in the NCBI SRA database under the study accession: PRJNA438041 (SRP136989).

The differentially expressed transcripts belonged in many cancer-related pathways. The signaling pathways (KEGG) activated by BPA in the HCT116 cell line are the Rap1 signaling pathway, signaling pathways regulating the pluripotency of stem cells, vascular smooth muscle contraction, the prolactin signaling pathway, the sphingolipid signaling pathway, the neurotrophin signaling pathway, and the cAMP signaling pathway. The shared gene ontology (GO) terms regulated by BPA in the HCT116 cell line are the positive regulation of GTPase activity, RNA splicing, regulation of RNA splicing, mRNA processing, positive regulation of DNA binding, regulation of small GTPase-mediated signal transduction, peptidyl-tyrosine phosphorylation, cell migration, innate immune response, transcription, DNA-templated, protein autophosphorylation, and positive regulation of lamellipodium morphogenesis.

The signaling pathway (KEGG) activated by BPA in the HCoEpiC cell line is the PPAR signaling pathway and the shared GO terms are the response to hypoxia, negative regulation of cell proliferation, cell cycle, positive regulation of angiogenesis, positive regulation of transcription from RNA polymerase II promoter, signal transduction, peptidyl-serine phosphorylation, cellular response to transforming growth factor beta stimulus, negative regulation of cell-cell adhesion mediated by cadherin, response to estrogen, positive regulation of endothelial cell proliferation, positive regulation of chemokine biosynthetic process, hepatocyte apoptotic process, negative regulation of blood vessel endothelial cell migration, intermediate filament cytoskeleton organization, cellular response to prostaglandin E stimulus, positive regulation of mitotic nuclear division, and negative regulation of apoptotic process.

### 2.6. Confirmation of Differentially Expressed Genes by qRT-PCR

**Figure 5:**
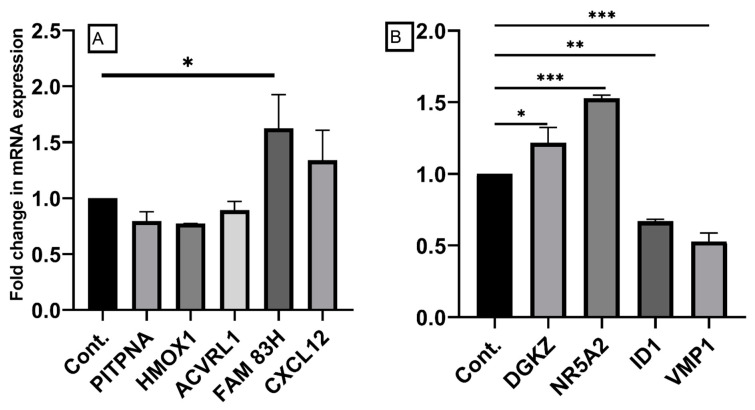
Confirmation of the differentially expressed genes by qRT-PCR. The figure shows the normalized relative expression of the test gene vs. the vehicle (DMSO)-exposed control. (**A**) The HCoEpiC cells, and (**B**) the HCT116 cells. Gene expression was determined using real-time quantitative reverse transcription PCR. Values are represented as means ± SEMs, with *n* = 3 (3 biological × 3 technical). * *p* < 0.05, ** *p* < 0.01, and *** *p* < 0.001.

We selected some of the most significant differentially expressed genes for confirmation by qRT-PCR. The selection was based upon a high fold change and the gene’s potential role in carcinogenesis. For the HCoEpiC cells, we selected *PITPNA* and *HMOX1* from the under-expressed genes and *FAM83H*, *CXCL12*, and *ACVRL1* from the over-expressed genes. Overall, the pattern of expression was consistent with what was expected from the NGS data for all but *ACVRL1*. For the HCT116 cells, we selected *DGKZ* and *NR5A2* from the over-expressed genes and *VMP1* and *ID1* from the downregulated genes. The pattern of expression of these genes was consistent with what was expected from the NGS data (Figure 5).

## 3. Discussion

BPA is a common environmental contaminant that usually gets in the human body via ingestion. There is clinical evidence of increased exposure of colorectal cancer patients to BPA [19,20]. However, the exact role and mechanism of action of BPA in colon cancer development and progression still need to be clarified. In the current work, we showed that BPA can induce changes in the colon epithelial cells that are consistent with transformation, and we identified multiple molecular alterations and pathways associated with PBA oncogenicity in the colon.

We found that BPA was toxic to both colon cancer cells and normal colon epithelial cells, which is consistent with the literature [14,15,22]. Wang et al. showed that dietary BPA induced apoptosis and mitochondrial dysfunction in mouse colon and liver [14]. Of note is that BPA increased the levels of oxidative stress indicators such as ROS, RNS, and hydrogen peroxide in mouse colon [14,15], which might create a state of chronic inflammation and trigger carcinogenesis with prolonged exposure [20,23].

The prolonged exposure of colon epithelial cells to a low nanomolar dose of BPA increased cellular invasiveness through collagen and endowed cells with the ability to anchorage-independent cell growth. Both of these findings support BPA’s oncogenicity in the colon. Our data are consistent with the data of Jun et al., who found that BPA increased HT29 cell proliferation and migration via the phosphorylation of ERK, and it reduced E-cadherin expression, which can explain the increased migration ability. BPA also induced increased 5-HT3 receptor expression, a major mitogenic factor, which could explain the increased proliferation [24]. Shi et al. reported that BPA exposure promoted the migration of normal colon epithelial cells (NCM460) via the estrogen receptor-mediated integrin β1/matrix metalloproteinase 9 (MMP-9) pathway [25]. An earlier work showed that BPA increased the migration and invasion of SW480 colorectal cancer cells via the epithelial to mesenchymal transition (EMT) associated with the increased expression of N-cadherin and decreased E-cadherin and the stabilization of transcription factor SNAIL. The stabilization of SNAIL was due to AKT/GSK-3β-signaling, which is consistent with our phosphokinase data (discussed later) [26].

To gain insights into the mechanism of these actions, we performed transcriptomic analysis using NGS, which revealed 340 differentially expressed transcripts by BPA in the HCT116 cells and 75 in the HCoEpiC cells. These transcripts belong in many cancer-related pathways such as apoptosis, cell proliferation, signal transduction, and angiogenesis. The dysregulation of significant cancer-related genes (*FAM83H*, *CXCL12*, *PITPNA*, *HMOX1*, *DGKZ*, *NR5A2*, *VMP1*, and *ID1*) was confirmed by qRT-PCR. *FAM83H* is important in the development of teeth and it is believed to exert an oncogenic function because it is over-expressed in cancer tissues compared to its non-neoplastic counterparts [27]. The high expression of *FAM83H* is associated with poor prognoses and short survival rates in cancer patients, including those with gastrointestinal tract cancers such as gastric cancer [28]. FAM83H translocates to the nucleus in cancer cells where it acts in conjunction with MYC and the Wnt/β-catenin pathway to stimulate cell proliferation and induce EMT. Thus, it can explain the increased invasiveness of colon cells after BPA exposure. The deregulated nuclear localization of FAM83H has been reported in colorectal carcinoma [29] and was associated with short cancer-specific survival rates [30]. The C-X-C motif chemokine ligand 12 (*CXCL12*) promotes cancer progression in several models, including colorectal cancer [31], facilitates cancer metastasis, and is associated with poor prognoses in colorectal cancer patients [32]. The phosphatidylinositol transfer protein alpha (*PITPNA*) gene encodes a member of a family of lipid-binding proteins that transfer molecules of phosphatidylinositol or phosphatidylcholine between membrane surfaces, and it is implicated in phospholipase C-signaling. It is associated with favorable prognoses in many cancers, while its antisense long, noncoding RNA (lncRNA), *PITPNA-AS1*, is overexpressed in gastric cancer tissues and associated with poor survival rates in gastric cancer patients [33]. The heme oxygenase 1 gene (*HMOX1*) reduces oxidative stress and exerts diverse functions in cancer cells. However, a study reported that it inhibited TGF-β-induced EMT in MCF7 breast cancer cells [34]. Thus, its downregulation by BPA could be consistent with the observed increase in cell invasion in the current work. Diacylglycerol kinase zeta (*DGKZ*) was shown to promote TGF-β signaling in triple-negative breast cancer [35]. *DGKZ* also promotes Rho GTPase activity and contributes to enhanced invasion in metastatic colon cancer cells [36]. The nuclear receptor subfamily 5 group A member 2 (*NR5A2*), also known as liver receptor homolog-1 (*LRH-1*), encodes a transcription factor that regulates the expression of various oncogenes. *NR5A2* is over-expressed in pancreatic cancer and promotes EMT [37]. *NR5A2* and the key gene vimentin (*VIM*) were significantly co-expressed in cervical squamous cell carcinoma, and *VIM* was also significantly co-expressed with EMT signaling pathway genes [38]. Vacuole membrane protein 1 (*VMP1*) is an endoplasmic reticulum-resident and multi-spanning membrane protein. It plays an essential role in autophagy and its depletion substantially disrupts autophagosomes. There are contrasting reports on its role in different cancers. For example, it contributed to glioma development by regulating autophagy [39], while it exerted tumor suppressor features in colorectal cancer [40]. *VMP1* showed increased expression in non-cancer-adjacent tissues compared with that in colorectal cancer tissues. Its expression was decreased in the late stages of colorectal cancer. The median survival rate of patients with low *VMP1* expression was much shorter than the survival rate of patients with high expression. *VMP1* knockout in colon cancer cells promoted cell proliferation and invasion [40]. Finally, the inhibitor of DNA binding, *ID1*, which is an inhibitor of the basic helix-loop-helix transcription factor, can exert a wide range of functions in various types of tumors. *ID1* is a stem-cell-like gene that is over-expressed in numerous types of cancers, and it facilitates tumor angiogenesis and metastasis. However, controversial results have also been obtained [41]. For example, *ID1* inhibited EMT via disrupting the interaction between TCF4 and TWIST1 in lung epithelial cancer cells [42]. Taken together, the majority of the genes dysregulated by BPA in colon epithelial cells augment EMT, which is the most dominant phenotype in colorectal cancer [43], and they contribute to the proliferation, invasion, and metastasis of cancer cells and/or the resistance to therapy.

We performed comprehensive protein kinase phosphorylation analysis, which showed that BPA induced the phosphorylation of a few protein kinases such as JNK1/2/3, GSK-3α/β, AMPKα1, AKT1/2/3, AMPKα2, HSP27, β-catenin, STAT2, Hck, chk2, FAK, and PRAS40 in the HCoEpiC cells, and GSK-3α/β, p53, AKT1/2/3, p70 S6 kinase, and WNK1 in the HCT116 cells. A group of these kinases are involved in the DNA damage response (p53 and Chk2) and/or the stress response (JNK1/2/3, AMPKα1, AMPKα2, and HSP27). However, HSP27 can play a role in carcinogenesis under certain conditions [44]. The majority of the kinases that were phosphorylated by PBA are involved in potential carcinogenic pathways. Of particular significance is the activation of AKT, a serine/threonine kinase previously known as protein kinase B (PKB), consisting of three isoforms (Akt1, Akt2, and Akt3). It is a main hub that controls cell death and survival, and it plays a pivotal role in multiple interconnected signaling mechanisms, including cell metabolism, growth and division, apoptosis evasion, and angiogenesis [45]. Disruption of the AKT-regulated pathways is a common finding in many cancers, particularly colorectal cancer. Recent data have demonstrated a potentiating effect when both the PI3K/Akt and Wnt/β-catenin pathways were activated simultaneously with IGF-1 and Wnt3a, leading to enhanced cellular migration and proliferation [46]. β-catenin, an important transcription factor that triggers colorectal cancer proliferation and invasion via EMT, was found to be activated by BPA in the current work, too. Additionally, some other kinases that were found to be phosphorylated by BPA exposure (GSK-3α/β, Hck, FAK, and WNK1) contribute to increased cell migration and invasion via EMT. GSK-3 is a common kinase involved in the regulation of many oncogenic pathways, including Wnt/β-catenin and PI3K/PTEN/Akt/mTORC, and its deregulation by inhibitory phosphorylation results in EMT and cancer progression [47]. Consistent with our findings, Chen et al. reported that AKT/GSK-3β signaling was activated by BPA in colon cancer cells and resulted in the stabilization of the SNAIL transcription factor and EMT induction [26]. FAK is a cytoplasmic tyrosine kinase that plays a critical role in integrin-mediated signal transduction. Activated FAK signaling in colorectal cancer stimulates migration via EMT [48]. WNK1 is an atypical serine/threonine protein kinase that regulates ion homeostasis. Recent data have shown that WNK1 signaling pathways are additionally involved in angiogenesis, cancer proliferation, and metastasis. WNK1 can regulate many oncogenic pathways, including the WNT/β-catenin and TGF-β-SMAD2 pathways, and it can increase the nuclear levels of the EMT-related transcription factors SLUG and FOXO1 [49]. Detailed discussion of the oncogenic potential of other kinases that were phosphorylated by BPA can be found elsewhere [7,50,51].

## 4. Materials and Methods

### 4.1. Cell Lines

The normal colon epithelial cell HCoEpiC (from Science Cell Research Laboratories, Carlsbad, CA, USA) was initially cultured in its supplied special growth medium as per the supplier’s instructions. It was then maintained for the purpose of this experiment in DMEM/F12 ham, while the colon cancer cell line HCT116 was grown in DMEM. The culture media were supplemented by 10% charcoal stripped-fetal bovine serum and 1x penicillin and streptomycin. All cells were maintained at 37 °C in a humidified 5% CO2 incubator and were split when their confluency reached 70–90%, according to the suppliers’ instructions.

### 4.2. Toxic Chemicals and Cytotoxicity Assay

BPA was purchased from Sigma-Aldrich Corp. (St. Louis, MO, USA). The BPA was dissolved in 100% dimethyl sulfoxide (DMSO) and stored as stock solutions at 4 °C. The stocks of the BPA-exposed cells were frozen down in liquid nitrogen after one and two months. Initially, we performed a pilot study to find out the effect of increasing the doses of BPA on viability. Based upon our findings, we selected the dose of 0.0043 nM for further experiments. The highest DMSO concentration did not exceed 1%. The viability of the cells exposed to the toxic agents was determined using the MTT method. Briefly, cells were seeded in a 96-well plate (5 × 103 cells/well) and incubated with BPA or the vehicle for 48 h. After treatment, MTT solution was added to the medium and the cells were incubated at 37 °C for 2 h. The solubilization of the MTT crystals was accomplished by adding 100 μL of DMSO, followed by a 10 min incubation. Absorbance was measured using a microtiter plate reader at 570 nm. The rate of proliferation was calculated by comparing the absorption of the treated cultures with that of the untreated control cultures.

### 4.3. Invasion Assay

For the invasion assay, we used QCMTM High Sensitivity Non-cross-linked Collagen Invasion Assay kit, which was available from Millipore (Burlington, MA, USA), and followed the manufacturer’s protocol. Briefly, the assay was performed using a modified chamber with filter inserts (pore size of 8 μm) coated with matrigel in 24-well dishes. Approximately 0.5 million cells were prepared in the corresponding serum-free media. Two hundred and fifty microliters of the cell suspension were added into the inserts (top chamber) and 500 μL of 15% FBS-containing media were added to the bottom chamber. After a 48 h incubation, the cells remaining in the top chamber were removed and 400 μL of cell stain was applied to the invasion chamber insert for 15 min. After several washes with water, the inserts were dried and then transferred into 200 μL of extraction buffer and allowed to incubate for 15 min at room temperature. The dye mixture was then assessed by a plate reader at a wavelength of 560 nm.

### 4.4. Colony Formation in Soft Agar

The colony formation was analyzed using the commercial kit “Cell Transformation Detection Assay” (ECM570, CHEMICON, Millipore (Burlington, MA, USA)). The pre-treated cells with BPA or control cells were cultured in soft agar medium for 21–28 days. HeLa was used as a reference/positive control, while the DMSO-exposed cells were used as a negative control. The formed colonies were stained using a cell stain solution then counted morphologically.

### 4.5. Next-Generation Sequencing Analysis

RNA sequencing libraries were prepared with an Illumina-compatible NEBNext^®^ Ultra™ Directional RNA Library Prep Kit (New England BioLabs, MA, USA) at Genotypic Technology Pvt. Ltd., Bangalore, India via a local dealer (Genetrics, Dubai, UAE). Two hundred ng of total RNA were taken for mRNA isolation, fragmentation, and priming. The fragmented and primed mRNA were further subjected to first-strand synthesis in the presence of actinomycin D (Gibco, life technologies, CA, USA), followed by second-strand synthesis. The double-stranded cDNA was purified using HighPrep magnetic beads (Magbio Genomics Inc, USA). The purified cDNA was end-repaired, adenylated, and ligated to Illumina multiplex barcode adapters as per the NEBNext^®^ Ultra™ Directional RNA Library Prep Kit protocol. The Illumina Universal Adapters used in the study were: 5′-AATGATACGGCGACCACCGAGATCTACACTCTTTCCCTACACGACGCTCTTCCGATCT-3′ and the Index Adaptor: 5′-GATCGGAAGAGCACACGTCTGAACTCCAGTCAC [INDEX] ATCTCGTATGCCGTCTTCTGCTTG-3′. The [INDEX]—Unique sequence was used to identify sample-specific sequencing data. The adapter-ligated cDNA was purified using HighPrep beads and then subjected to 14 cycles of “Indexing-PCR” (37 °C for 15 min, followed by denaturation at 98 °C for 30 s, cycling (98 °C for 10 s, 65 °C for 75 s) then 65 °C for 5 min) to enrich the adapter-ligated fragments. The final PCR product (sequencing library) was purified with HighPrep beads, followed by a library quality control check. The Illumina-compatible sequencing library was quantified by a Qubit fluorometer (Thermo Fisher Scientific, MA, USA), and its fragment size distribution was analyzed on an Agilent 2200 TapeStation (Santa Clara, CA, USA).

### 4.6. Real Time-Polymerase Chain Reaction (RT-PCR)

RNA extraction: mRNA was extracted using an RNAeasy Mini Kit (Qiagen, Hilden, Germany). The cells were harvested when they reached 80% confluency. The cells were washed twice with warm PBS and trypsinized, then centrifuged for 5 min at 1100 rpm. The pellet was resuspended in 350 µL of RLT buffer. A similar volume of 70% ethanol was added. The total volume was transferred to an RNase spin column placed in a 2 mL collecting tube. The column was centrifuged for 15 s at >10,000 rpm at room temperature, the flow through was discarded, and the following different buffers were added: 700 µL of washing buffer (RW1), followed by 500 µL of RPE buffer, and every time, the column was centrifuged for 15 s at >10,000 rpm at room temperature. A total of 500 µL of RPE buffer was added and column was centrifuged 11,000 rpm for 2 min. The total mRNA was collected by adding 30–50 µL of RNase-free to the column membrane, then centrifuging for 1 min at 11,000 rpm. The RNA concentration was measured using a Nanodrop (Colibri Microvolume Spectrometer from Titertek-Berthold) by direct absorbance at A280. The collected RNA extraction was kept for long-term storage at −80 °C.

Reverse Transcription: cDNA was synthetized using a QuantiTect Reverse Transcription Kit (Qiagen, Hilden, Germany). A total of 1 µg of RNA was mixed with 2 μL of gDNA wipeout buffer, and Rnase-free water was added to the final volume of 14 μL. Then, the samples were heated for 2 min at 42 °C and 1 μL of Quantiscript reverse transcriptase, 1 μL RT primer mix, and 4 μL Quantiscript RT buffer were added to each of resultant sample from the previous step. The samples were heated for 15 min at 42 °C and then for 3 min at 95 °C. The cDNA was kept in storage at −20 °C.

qRT-PCR: qRT-PCR was performed using 5x HOT FIREPol^®^ EvaGreen^®^ qPCR Mix Plus (ROX) (Solis BioDyne, Tartu, Estonia). The mRNA expression levels were amplified using a QuantStudio3 system. GAPDH was used as an internal control, and 2-ɅɅCT was used to calculate the mRNA’s relative expression. Table 1 summarize the primers and their sequences used in this study.

### 4.7. Proteome Profile/Human Phospho-Kinase Array

We analyzed the alterations of kinase signaling in response to the two-month exposure to BPA using a “human phospho-kinase array” ready-made kit (Proteome Profiler; R&D Systems, Minneapolis, MN, USA). The array analyzes 43 kinase phosphorylation sites and 2 related total proteins with carefully selected specific capture antibodies. The experimental technique and analyses were conducted according to the manufacturer’s guidelines, which were found in the booklet that also contained a list of the targets and phosphorylation sites, as described earlier [52].

### 4.8. Data Analysis

We used the Student t-test for significance and/or one-way ANOVA, followed by a post hoc test for the statistical analysis of the normally distributed data. The data are expressed as means ± the SDs of 3 biological repetitions. The graphs were generated using GraphPad Prism version 9.0.0.(121). A *p* value of < 0.05 was considered to determine statistical significance.

## 5. Conclusions

Taken together, these data showed that BPA plays a role in colon cancer development and progression, and they provide insights into the molecular mechanism of colon epithelial cell transformation by BPA. It is remarkable that many of the genes and kinases dysregulated by BPA exposure can trigger cell proliferation and invasion via EMT, which is a dominant phenotype of colorectal cancer. Increasing exposure to environmental toxins such as BPA can explain the increasing incidence of colorectal cancer. Therefore, the preventive measures for colorectal cancer should include limiting exposure to environmental toxins.

## Figures and Tables

**Table 1:** Sequence of primers used for qRT-PCR in this study.

FAM83H	Forward: GAAGTTCCTGCTGGTGGACTGT
	Reverse: AGACCAGCTCTCCTTGGAACAC
CXCL12	Forward: CTCAACACTCCAAACTGTGCCC
	Reverse: CTCCAGGTACTCCTGAATCCAC
ACVRL1	Forward: GCGACTTCAAGAGCCGCAATGT
	Reverse: TAATCGCTGCCCTGTGAGTGCA
PITPNA	Forward: GCAGATCGAAGCCAAGTGCTCA
	Reverse: GGCAGTCCTTCTGGTTTACAAGC
HMOX1	Forward: CCAGGCAGAGAATGCTGAGTTC
	Reverse: AAGACTGGGCTCTCCTTGTTGC
DGKZ	Forward: GCTTTCTCTGACTTCCTGATGGG
	Reverse: GGTTTCAGGTCCTGGATCTTGG
NR5A2	Forward: GGCTTATGTGCAAAATGGCAGATC
	Reverse: GCTCACTCCAGCAGTTCTGAAG
VMP1	Forward: TTGGAACAGGGCTGCACACCTT
	Reverse: TCAGGATAGGGTGGTTCGGGAA
ID1	Forward: GTTGGAGCTGAACTCGGAATCC
	Reverse: ACACAAGATGCGATCGTCCGCA
GAPDH	Forward: GTCTCCTCTGACTTCAACAGCG
	Reverse: ACCACCCTGTTGCTGTAGCCAA

## Data Availability

The NGS data have been deposited in the NCBI SRA database under study accession number PRJNA438041 (SRP136989).

## Supplementary Materials

Supporting information can be downloaded at: https://www.mdpi.com/article/10.3390/ijms231911620/s1.
